# Supplementary material for: Methods Used to Evaluate the Immediate Effects of Airway Clearance Techniques in Adults with Cystic Fibrosis: A Systematic Review and Meta-Analysis
Source: J Clin Med. 2021 Nov 13;10(22):5280. doi: 10.3390/jcm10225280 (PMC8625729; doi:10.3390/jcm10225280)
Supplement: Supplementary file 1 [file jcm-10-05280-s001.zip › jcm-1419105-supplementary.pdf]

Methods used to evaluate the immediate effects of airway clearance techniques in adults with cystic fibrosis: a systematic review and meta-analysis.

Naomi Chapman[1,2,3], Kathryn Watson[2], Tamara Hatton[2], Vinicius Cavalheri[1,3,4,5*], Jamie Wood[6], Daniel F. Gucciardi[1,4], Elizabeth F. Smith[7], and Kylie Hill[1,3,4]

1 [1] Curtin School of Allied Health, Faculty of Health Sciences, Building 401 Curtin University, Kent St, Bentley 6102, Australia; naomi.chapman2@health.wa.gov.au (N.C.); d.gucciardi@curtin.edu.au (D.F.G.); k.hill@curtin.edu.au (K.H.)

2 Physiotherapy Department, Sir Charles Gairdner Hospital, Hospital Ave, Nedlands 6009, Australia; kathryn.watson@health.wa.gov.au (K.W.); tamara.hatton@health.wa.gov.au (T.H.)

3 Institute for Respiratory Health, 6 Verdun St, Nedlands 6009, Australia

4 Curtin enAble Institute, Faculty of Health Sciences, Building 408 Curtin University, Kent St, Bentley 6102, Australia

5 Allied Health, South Metropolitan Health Service, 11 Robyn Warren Dr, Murdoch 6150, Australia

6 Abilities Research Center, Department of Rehabilitation and Human Performance, Icahn School of Medicine at Mount Sinai, New York City, New York 10029, USA; jamie.wood@mountsinai.org

7 Children's Lung Health Team, Wal-Yan Respiratory Research Centre, Telethon Kids Institute, Perth Children's Hospital, 15 Hospital Av, Nedlands 6009, Australia; elizabeth.smith@telethonkids.org.au

* Correspondence: vinicius.cavalheri@curtin.edu.au

Keywords: Cystic Fibrosis; Adults; Airway Clearance; Chest Physiotherapy; Outcome measures

Abbreviations: ACT: airway clearance techniques, C: cough alone, COPD: chronic obstructive pulmonary disease, MCID: minimally clinical important differences, FEV_1: forced expiratory volume in one second, MBW: multiple breath washout, NIV: non-invasive ventilation, OT: other techniques, PEP: positive expiratory pressure device, PPD: positive-pressure devices, RCT: randomised controlled trials, RV: residual volume, RXT: randomised cross-over trials, TIDieR: Template for Intervention Description and Replication

METHODS

Data analysis

For measures collected during and/or after completion of the ACT (but not prior to ACT, such as sputum weight), data reported within those 60 minutes after ACT were used in the meta-analysis. If multiple time points were collected within that 60 minutes, only the time point closest to 60 minutes was used. For measures collected before and after ACT (e.g. forced expiratory volume in one second [FEV_1]) the mean change and, where possible, the SD of the mean change was used. If the SD of the mean change was unavailable, the SD of the measures collected prior to ACT was used. Values reported as median, IQR or ranges of skewed data were transformed to mean and SD using an online software [1]. If only standard error of the mean was reported then this was back converted using the RevMan calculator to mean and SD [2]. When analysing between-group changes, studies with two or more intervention groups for which the mechanism of action of the ACT performed was the same, a conservative approach was taken, and data from the intervention group with the lowest value were used.

RESULTS

Study characteristics

Figure S1: Risk of Bias analyses

(a) Randomised crossover trials

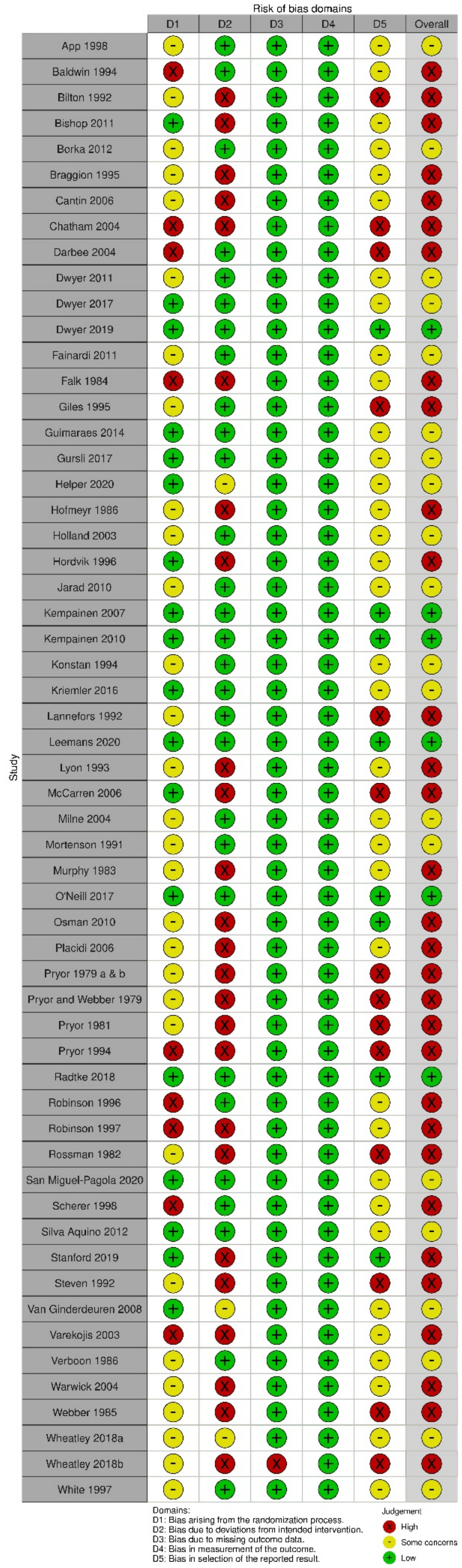

(b) Randomised controlled trials

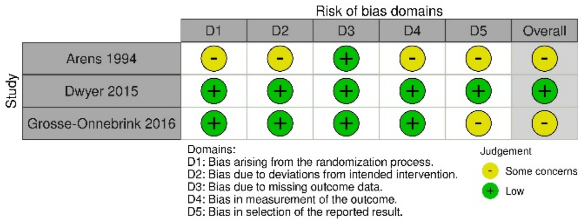

Magnitude of between-group change following ACT

For sputum wet weight, 14 of the 65 studies (22%) provided adequate data to be included in the meta-analyses of sputum wet weight. Comparisons were possible for (i) PEP *versus* C (Figure S2a; five studies), and (ii) OT *versus* C (Figure S2b; five studies). No between-group difference in sputum wet weight was demonstrated in any of the comparisons. Due to substantial heterogeneity in the meta-analyses that compared PEP *versus* C (Figure S2a; I^2 = 89%) and OT *versus* C (Figure S2b; I^2 = 88%), a sensitivity analysis was undertaken. The studies by Mortensen et al [3] and Konstan et al [4] were omitted due to substantial difference in the mean number of productive coughs between treatment groups (PEP = 13.5 coughs; OT = 11.5 coughs; and C = 1 cough) [3], or discrepancies in the amount of coughing that was encouraged between the different groups [4]. The sensitivity analyses reduced heterogeneity (I^2 = 0% for all), but did not change the finding of no between-group difference (PEP *versus* C, 0.34g [-1.14; 1.82], OT *versus* C, 0.49g [-1.07; 2.04]). The results for the between-group comparison using sputum dry weight and FEV_1 can be seen in the following figures S3a (PEP *versus* C), b (PEP *versus* C sensitivity analyses) and c (OT *versus* C) and S4a (PEP *versus* C) respectively. In summary, there were no between-group differences in sputum dry weight or FEV_1 demonstrated in any of the comparisons with cough alone even when sensitivity analyses were completed as per sputum wet weight ($p>0.05$ for all).

Figure S2. Between-group differences sputum wet weight **(a)** PEP *versus* C, **(b)** OT *versus* C; C = cough alone, OT = other techniques, PEP = positive expiratory pressure device

(a)

	PEP			Cough				Mean difference	Risk of Bias					
Study or Subgroup	Mean [Grams]	SD [Grams]	Total	Mean [Grams]	SD [Grams]	Total	Weight	IV, Random, 95% CI [Grams]	A	B	C	D	E	F
✓ Mortensen 1991	10.15	5.301	10	0.525	0.678893	10	19.7%	9.63 [6.31 , 12.94]	?	+	+	+	?	?
✓ Konstan 1994	10.4	6.7	17	2.7	2.7	17	19.5%	7.70 [4.27 , 11.13]	?	+	+	+	?	?
✓ Braggion 1995	2.61	2.66	16	2.22	2.86	16	21.6%	0.39 [-1.52 , 2.30]	?	-	+	+	?	-
✓ Placidi 2006	15.78	5.49	17	13.98	4.96	17	19.3%	1.80 [-1.72 , 5.32]	?	-	+	+	?	-
✓ Jarad 2010	2.086667	4.504963	18	3.053333	5.092218	18	19.9%	-0.97 [-4.11 , 2.17]	?	+	+	+	?	?
**Total (95% CI)**			**78**			**78**	**100.0%**	**3.63 [-0.34 , 7.60]**						

Heterogeneity: Tau² = 18.04; Chi² = 36.02, df = 4 (P < 0.00001); I² = 89%
Test for overall effect: Z = 1.79 (P = 0.07)
Test for subgroup differences: Not applicable

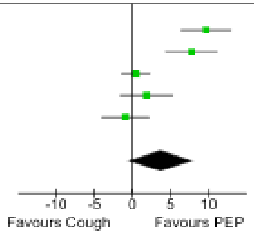

Risk of bias legend
(A) Bias arising from the randomization process
(B) Bias due to deviations from intended interventions: Sputum wet weight
(C) Bias due to missing outcome data: Sputum wet weight
(D) Bias in measurement of the outcome: Sputum wet weight
(E) Bias in selection of the reported result: Sputum wet weight
(F) Overall bias: Sputum wet weight

(b)

	OT			Cough				Mean difference	Risk of Bias					
Study or Subgroup	Mean [Grams]	SD [Grams]	Total	Mean [Grams]	SD [Grams]	Total	Weight	IV, Random, 95% CI [Grams]	A	B	C	D	E	F
✓ Mortensen 1991	8.05	3.75	10	0.525	0.679	10	20.9%	7.53 [5.16 , 9.89]	?	+	+	+	?	?
✓ Steven 1992	35.10493	7.538	24	33.3	5.2769	24	17.8%	1.80 [-1.88 , 5.49]	?	-	+	+	-	-
✓ Konstan 1994	2.2	2.2	17	2.7	2.7	17	22.2%	-0.50 [-2.16 , 1.16]	?	+	+	+	?	?
✓ Braggion 1995	2.47	2.65	16	2.22	2.86	16	21.8%	0.25 [-1.66 , 2.16]	?	-	+	+	?	-
✓ Jarad 2010	3.053333	6.676999	18	3.053333	5.092218	18	17.4%	0.00 [-3.88 , 3.88]	?	+	+	+	?	?
**Total (95% CI)**			**85**			**85**	**100.0%**	**1.83 [-1.29 , 4.96]**						

Heterogeneity: Tau² = 10.76; Chi² = 32.83, df = 4 (P < 0.00001); I² = 88%
Test for overall effect: Z = 1.15 (P = 0.25)
Test for subgroup differences: Not applicable

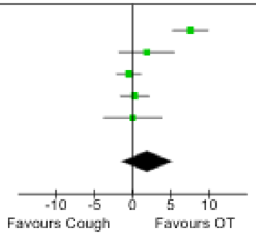

Risk of bias legend
(A) Bias arising from the randomization process
(B) Bias due to deviations from intended interventions: Sputum wet weight
(C) Bias due to missing outcome data: Sputum wet weight
(D) Bias in measurement of the outcome: Sputum wet weight
(E) Bias in selection of the reported result: Sputum wet weight
(F) Overall bias: Sputum wet weight

Figure S3. Between-group differences sputum dry weight **(a)** PEP *versus* C, **(b)** PEP *versus* C sensitivity analyses, **(c)** OT *versus* C; C = cough alone, OT = other techniques, PEP = positive expiratory pressure device, PPD = positive pressure devices

(a)

	PEP			Cough				Mean difference	Risk of Bias					
Study or Subgroup	Mean [Grams]	SD [Grams]	Total	Mean [Grams]	SD [Grams]	Total	Weight	IV, Random, 95% CI [Grams]	A	B	C	D	E	F
✓ Konstan 1994	8.2	7.2	17	1.7	2.1	17	1.0%	6.50 [2.93 , 10.07]	?	+	+	+	?	?
✓ Braggion 1995	0.47	0.33	16	0.41	0.37	16	35.0%	0.06 [-0.18 , 0.30]	?	-	+	+	?	-
✓ Placidi 2006	0.94	0.57	17	0.97	0.76	17	25.2%	-0.03 [-0.48 , 0.42]	?	-	+	+	?	-
✓ Jarad 2010	0.1	0.217204	18	0.12	0.241337	18	38.8%	-0.02 [-0.17 , 0.13]	?	+	+	+	?	?
**Total (95% CI)**			**68**			**68**	**100.0%**	**0.07 [-0.29 , 0.43]**						

Heterogeneity: Tau² = 0.08; Chi² = 13.07, df = 3 (P = 0.004); I² = 77%
Test for overall effect: Z = 0.38 (P = 0.70)
Test for subgroup differences: Not applicable

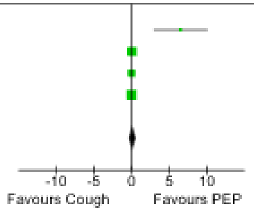

Risk of bias legend
(A) Bias arising from the randomization process
(B) Bias due to deviations from intended interventions: Sputum dry weight
(C) Bias due to missing outcome data: Sputum dry weight
(D) Bias in measurement of the outcome: Sputum dry weight
(E) Bias in selection of the reported result: Sputum dry weight
(F) Overall bias: Sputum dry weight

(b)

	PEP			Cough				Mean difference	Risk of Bias					
Study or Subgroup	Mean [Grams]	SD [Grams]	Total	Mean [Grams]	SD [Grams]	Total	Weight	IV, Fixed, 95% CI [Grams]	A	B	C	D	E	F
✕ Konstan 1994	8.2	7.2	17	1.7	2.1	17	0.0%	6.50 [2.93 , 10.07]	?	+	+	+	?	?
✓ Braggion 1995	0.47	0.33	16	0.41	0.37	16	25.6%	0.06 [-0.18 , 0.30]	?	-	+	+	?	-
✓ Placidi 2006	0.94	0.57	17	0.97	0.76	17	7.4%	-0.03 [-0.48 , 0.42]	?	-	+	+	?	-
✓ Jarad 2010	0.1	0.217204	18	0.12	0.241337	18	67.0%	-0.02 [-0.17 , 0.13]	?	+	+	+	?	?
**Total (95% CI)**			**51**			**51**	**100.0%**	**-0.00 [-0.12 , 0.12]**						

Heterogeneity: Chi² = 0.32, df = 2 (P = 0.85); I² = 0%
Test for overall effect: Z = 0.00 (P = 1.00)
Test for subgroup differences: Not applicable

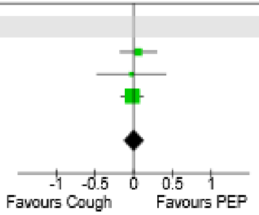

Risk of bias legend
(A) Bias arising from the randomization process
(B) Bias due to deviations from intended interventions: Sputum dry weight
(C) Bias due to missing outcome data: Sputum dry weight
(D) Bias in measurement of the outcome: Sputum dry weight
(E) Bias in selection of the reported result: Sputum dry weight
(F) Overall bias: Sputum dry weight

(c)

Study or Subgroup	OT Mean [Grams]	OT SD [Grams]	OT Total	Cough Mean [Grams]	Cough SD [Grams]	Cough Total	Weight	Mean difference IV, Fixed, 95% CI [Grams]
✓ Konstan 1994	1.8	2	17	1.7	2.1	17	0.9%	0.10 [-1.28 , 1.48]
✓ Braggion 1995	0.46	0.33	16	0.41	0.37	16	30.1%	0.05 [-0.19 , 0.29]
✓ Jarad 2010	0.11	0.24938191	18	0.12	0.24133733	18	69.0%	-0.01 [-0.17 , 0.15]
**Total (95% CI)**			**51**			**51**	**100.0%**	**0.01 [-0.12 , 0.14]**

Heterogeneity: Chi² = 0.18, df = 2 (P = 0.91); I² = 0%
Test for overall effect: Z = 0.13 (P = 0.89)
Test for subgroup differences: Not applicable

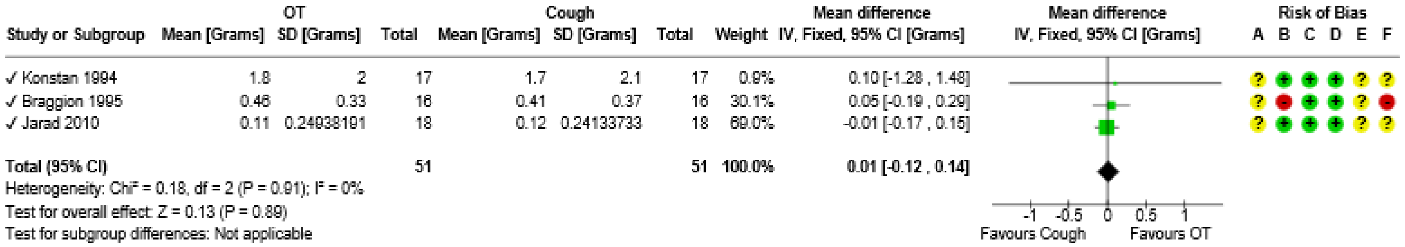

Risk of bias legend
(A) Bias arising from the randomization process
(B) Bias due to deviations from intended interventions: Sputum dry weight
(C) Bias due to missing outcome data: Sputum dry weight
(D) Bias in measurement of the outcome: Sputum dry weight
(E) Bias in selection of the reported result: Sputum dry weight
(F) Overall bias: Sputum dry weight

Figure S4. Between-group differences FEV1 in litres (PEP *versus* C); C = cough alone, FEV1 = forced expiratory volume in one second, OT = other techniques, PEP = positive expiratory pressure device

	PEP			Cough				Mean difference	Risk of Bias					
Study or Subgroup	Mean [Litres]	SD [Litres]	Total	Mean [Litres]	SD [Litres]	Total	Weight	IV, Fixed, 95% CI [Litres]	A	B	C	D	E	F
✓ Jarad 2010	-0.04	0.73	18	-0.01	0.58	18	14.6%	-0.03 [-0.46 , 0.40]	?	+	+	+	?	?
✓ Placidi 2006	0.01	0.27	17	0.03	0.26	17	85.4%	-0.02 [-0.20 , 0.16]	?	-	+	+	?	-
**Total (95% CI)**			**35**			**35**	**100.0%**	**-0.02 [-0.19 , 0.14]**						

Heterogeneity: Chi² = 0.00, df = 1 (P = 0.97); I² = 0%
Test for overall effect: Z = 0.26 (P = 0.80)
Test for subgroup differences: Not applicable

Mean difference
IV, Fixed, 95% CI [Litres]

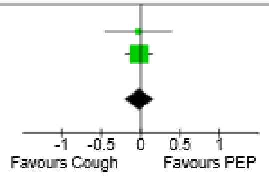

Risk of bias legend
(A) Bias arising from the randomization process
(B) Bias due to deviations from intended interventions: FEV1
(C) Bias due to missing outcome data: FEV1
(D) Bias in measurement of the outcome: FEV1
(E) Bias in selection of the reported result: FEV1
(F) Overall bias: FEV1

Supplement References:

1. Wan, X.; Wang, W.; Liu, J.; Tong, T. Estimating the sample mean and standard deviation from the sample size, median, range and/or interquartile range. *BMC Med Res Methodol* **2014**, *14*, 135, doi:10.1186/1471-2288-14-135.
2. *Review Manager Web (RevMan Web)*, Version 3.1.2.; The Cochrane Collaboration: 2021 Available online: https://revman.cochrane.org/#/myReviews (accessed on 3rd March 2021).
3. Mortensen, J.; Falk, M.; Groth, S.; Jensen, C. The effects of postural drainage and positive expiratory pressure physiotherapy on tracheobronchial clearance in cystic fibrosis. *Chest* **1991**, *100*, 1350-1357, doi:10.1378/chest.100.5.1350.
4. Konstan, M.W.; Stern, R.C.; Doershuk, C.F. Efficacy of the Flutter device for airway mucus clearance in patients with cystic fibrosis. *J Pediatr* **1994**, *124*, 689-693.
